# Race and Ethnicity, Deprivation, and Infant Mortality in England, 2019-2022

**DOI:** 10.1001/jamanetworkopen.2023.55403

**Published:** 2024-02-12

**Authors:** David E. Odd, Sylvia Stoianova, Tom Williams, Dawn Odd, Ngozi Edi-osagie, Charlotte McClymont, Peter Fleming, Karen Luyt

**Affiliations:** 1 Division of Population Medicine, School of Medicine, University of Cardiff, Cardiff, United Kingdom; 2 National Child Mortality Database, Bristol Medical School, University of Bristol, St Michael’s Hospital, Bristol, United Kingdom; 3 School of Health and Social Wellbeing, University of the West of England, Blackberry Hill, Bristol, United Kingdom; 4 Newborn Intensive Care Unit, Manchester University NHS Foundation Trust, Manchester, United Kingdom; 5 UCLPartners Academic Health Science Network, London, United Kingdom; 6 The National Institute for Health and Care Research Applied Research Collaboration West (NIHR ARC West) at University Hospitals Bristol and Weston NHS Foundation Trust, Bristol, United Kingdom

## Abstract

**Importance:**

England has one of the highest infant mortality rates in Europe. Much of the variation in infant mortality rates between races and ethnicities may be due to socioeconomic factors, but how deprivation and race and ethnicity are associated with infant mortality is unclear.

**Objectives:**

To investigate the association of infant race and ethnicity with the infant mortality rate in England, adjusted for preterm birth and level of deprivation.

**Design, Setting, and Participants:**

This cohort study included children who died younger than 1 year of age, born at or after 22 weeks’ gestation, occurring from April 1, 2019, to March 31, 2022, in England. Characteristics of the infant were derived from death notifications.

**Exposures:**

The racial and ethnic groups were derived from National Health Service data and were reported by the parents and characterized using the Office of National Statistics classification: Asian or Asian British (Bangladeshi, Chinese, Indian, Pakistani, or any other Asian background), Black or Black British (African, Caribbean, or any other Black background), multiracial (White and Asian, White and Black African, White and Black Caribbean, or any other multiracial background), White or White British (British, Irish, any other White background, or Gypsy or Irish Traveler), and other (Arab or any other racial or ethnic group).

**Main Outcomes and Measures:**

Risk of death for all racial and ethnic groups and relative risk of death compared with the reference group (White) were calcuated. Analyses were repeated, adjusting for deprivation, gestational age of infants, and region of England.

**Results:**

A total of 5621 infants who died younger than 1 year of age were reported to the National Child Mortality Database. A total of 2842 of 5130 infants (55.4%) were male; the median gestational age was 33 weeks (IQR, 25-38 weeks); of 5149 infants, 927 (18.0%) were Asian, 448 (8.7%) were Black, 3318 (64.4%) were White, 343 (6.7%) were multiracial, and 113 (2.2%) were from other racial and ethnic groups; and the median deprivation score was 4 (IQR, 3-5). In the unadjusted analysis, the relative risk of death compared with White infants was higher for Black (1.93 [95% CI, 1.75-2.13]) and Asian (1.67 [95% CI, 1.55-1.80]) infants. The population attributable risk fraction for all mortality rates among infants who were not White was 12.0% (95% CI, 10.3%-13.8%) (unadjusted), 9.8% (95% CI, 8.0%-11.7%) (adjusted for deprivation), 7.7% (95% CI, 5.9%-9.5%) (adjusted for gestational age at birth), and 12.8% (95% CI, 11.0%-14.5%) (adjusted for region of England).

**Conclusions and Relevance:**

This cohort study suggests that the proportion of infants who died before 1 year of age is associated with race and ethnicity, with a population attributable risk fraction of 12.0%. An overconservative adjustment for deprivation did not explain the overall patterns seen. Approximately half the population attributable risk fraction may be due to increased risk of preterm birth in Asian and Black communities. Work is needed to identify what can be done to reduce this incidence of infant mortality.

## Key Points

**Question:**

What is the association of race and ethnicity with the infant mortality rate in England?

**Findings:**

This cohort study included 5621 children who died younger than 1 year of age and found that, if all infants had the same risk of death as White infants, 12.0% of deaths could be avoided, bringing England to the European Union mean rate for infant mortality. Deprivation was not associated with the overall patterns seen.

**Meaning:**

This study suggests that, although deprivation did not explain the overall patterns seen, half the population attributable risk fraction for all infant mortality may be due to increased preterm births in Asian and Black communities.

## Teaser

This cohort study investigates the association of infant race and ethnicity with the infant mortality rate in England, adjusted for preterm birth and measures of deprivation.

## Introduction

England has one of the highest infant mortality rates in Europe,^1,2^ with much of the variation associated with socioeconomic factors.^3,4^ The National Healthcare Inequalities Improvement Programme introduced health policies to influence multiagency action on the social determinants of disease and address inequalities in health care provision, yet none of the policies have the explicit goal to reduce excess infant mortality in disadvantaged populations.^5,6,7^ Although approximately 65% of the child population in England is White, race and ethnicity vary across the regions of England and are patterned by deprivation level, making interpretation challenging,^4,8^ and the associations between these social measures and poor health outcomes are complex.^9,10^ Associations between social inequalities and death appear strongest for infant mortality,^11^ and it has been proposed that “ethnicity is not the major driver of health inequalities in the UK but deprivation, geography and differential exposure to key risk factors”^12^ are likely to underpin the associations seen.^9^ In addition, while absolute increases in infant mortality are recognized to be patterned by race and ethnicity, the causes of death and the likely mechanisms are unclear and likely to vary across social groups.^13^ The aim of this work is to investigate the association of the infant mortality rate (deaths at <1 year of age) in England with the race and ethnicity of the infants as well as how this infant mortality rate is associated with preterm birth, measures of deprivation, and the areas of England in which these infants lived.

## Methods

### Data, Study Design, and Population

The National Child Mortality Database (NCMD) commenced data collection on April 1, 2019. Data were downloaded on July 14, 2022. Data on the study population were derived from the Office of National Statistics (ONS) total births statistics (2019 birth characteristics data)^14^ and include a measure of race and ethnicity, stratified by deprivation measures, gestational age at birth, and region of England. Deaths of children younger than 1 year of age, born at 22 weeks’ gestation or older, occurring from April 1, 2019, to March 31, 2022, were identified. This cohort study followed the Strengthening the Reporting of Observational Studies in Epidemiology (STROBE) reporting guideline for observational studies. This work was reviewed by the Chair of the Central Bristol National Health Service (NHS) Research Ethics Committee, who confirmed that NHS ethical approval, including obtaining individual consent, was not required. The NCMD legal basis to collect confidential and personal level data under the Common Law Duty of Confidentiality has been established through the Children Act 2004 Sections M-N, Working Together to Safeguard Children 2018. The NCMD legal basis to collect personal data under the General Data Protection Regulation (GDPR) without consent is defined by GDPR Article 6 (e) Public task and 9 (h) Health or social care (with a basis in law). See the eMethods and eTable 1 in Supplement 1 for further details.

### Exposure

The racial and ethnic groups were derived from NHS data and were reported by the parents and characterized using the ONS classification: Asian or Asian British (Bangladeshi, Chinese, Indian, Pakistani, or any other Asian background), Black or Black British (African, Caribbean, or any other Black background), multiracial (White and Asian, White and Black African, White and Black Caribbean, or any other multiracial background), White or White British (British, Irish, any other White background, or Gypsy or Irish Traveler), and other (Arab or any other racial or ethnic group). When deriving relative risks (RRs), the White population of infants was considered as the reference group because it was the largest.

### Outcome

All deaths of children in England will ultimately be reviewed in depth by the Child Death Overview Panels, but this process often takes many months. In this work, similar to our previous work, to obtain a provisional category of death, all child deaths reported to the NCMD were coded by 3 independent coders (all pediatricians) to identify the most likely category of the cause of death.

### Confounders

The confounders investigated were the region of England where the death was reported, the gestational age of the child at birth (both reported by the Child Death Overview Panels at notification), and, from the child’s home postcode at the time of their death, the Index of Multiple Deprivation, a measure of local deprivation.^15^ This score ranges rom 1 to 10, with a lower value suggesting greater deprivation).

### Statistical Analysis

Statistical analysis is based on previous studies using similar data.^4,16,17,18^ Initially, we compared the characteristics of those infants by their reported race and ethnicity. A multiple imputation technique was performed to impute missing values of race and ethnicity, gestational age, provisional category of death, and deprivation. Datasets were collapsed down to the number of deaths in the estimated population at risk by racial and ethnic group and by risks, and RRs of death compared with White infants were derived assuming a Poisson regression model. To measure the association, the population attributable risk fraction (PAF) was derived, assuming that all infants had the same risk of death as White infants. The PAF estimates the reduction in deaths, across the whole population, that would be seen if all infants had the same risk of death as the comparison group.^19^ This process was repeated for each provisional category of death.

To test how much of the PAF was associated with the patterning of location, local environment, and perinatal events, analyses were then repeated, adjusting for deprivation decile (using the Index of Multiple Deprivation),^15^ gestational age at birth (in completed weeks), and region of England (using ONS categories). All 3 were added as categorical terms to the model and separately because granular data containing all 4 measures (race and ethnicity, deprivation, gestational age at birth, and region of England) were not available. Analysis of preterm deaths, adjusted for gestational age, was restricted to infants born preterm. The analysis was repeated for infants who died after May 1, 2021, of underlying conditions and was further presented and split by the additional subcategories available (cardiac [congenital], other chromosomal or congenital, or other [including acute medical condition, chronic medical conditions, cardiac (acquired), or other conditions]). As a sensitivity analysis, a complete case analysis was performed.

Data are presented as median number and percentage, risk per 100 000 person-years (95% CI), median number and IQR, or RR ratio (95% CI). All tests were 2-sided, and *P* < .05 was considered statistically significant. Analysis was performed using Stata, version 17 (StataCorp).

## Results

Over the 36 months, a total of 5621 infants who died younger than 1 year of age were reported to the NCMD (race and ethnicity data were unavailable for 472 infants [8.4%]). A total of 2842 of 5130 infants (55.4%) were male; the median gestational age was 33 weeks (IQR, 25-38 weeks); of 5149 infants, 927 (18.0%) were Asian, 448 (8.7%) were Black, 3318 (64.4%) were White, 343 (6.7%) were multiracial, and 113 (2.2%) were from other racial and ethnic groups (Table 1); and the median deprivation score was 4 (IQR, 3-5). London had more deaths than any other region (15.9% [895 of 5621]) (eTable 2 in Supplement 1). The suspected cause of death, the gestational age of the infants, the distribution across England, and the deprivation profile varied by race and ethnicity (Figure).

**Table 1.** Provisional Cause of Infant Deaths Reported to the National Child Mortality Database in England Between April 2019 and March 2022

Measure	No.	Infants, No./total No. (%)					*P* value
		White	Asian	Black	Multiracial	Other^a^	
Estimated No. of births	1 780 149	1 301 067/1 780 149 (73.1)	219 153/1 780 149 (12.3)	91 425/1 780 149 (5.1)	123 246/1 780 149 (6.9)	45 558/1 780 149 (2.6)	NA
All deaths	5149	3318/5149 (64.4)	927/5149 (18.0)	448/5149 (8.7)	343/5149 (6.7)	113. 5149 (2.2)	NA
Death by cause							
Malignant neoplasm	44	24/3239 (0.7)	11/901 (1.2)	5/439 (1.1)	NA	NA	<.001
Preterm birth	1944	1254/3239 (38.7)	330/901 (36.6)	202/439 (46.0)	125/338 (37.0)	33/111 (29.7)	
Intrapartum event	498	360/3239 (11.1)	62/901 (6.9)	32/439 (7.3)	32/338 (9.5)	12/111 (10.8)	
Infection	148	96/3239 (3.0)	26/901 (2.9)	12/439 (2.7)	11/338 (3.3)	NA	
Trauma	63	50/3239 (1.5)	NA	NA	NA	NA	
SUDIC	790	579/3239 (17.9)	72/901 (8.0)	50/439 (11.4)	75/338 (22.2)	14/111 (12.6)	
Underlying disease	1541	876/3239 (27.1)	396/901 (44.0)	134/439 (30.5)	89/338 (26.3)	46/111 (41.4)	

Abbreviations: NA, not applicable; SUDIC, sudden unexpected death in infancy and childhood.

^a^ Defined as Arab or any other racial and ethnic groups.

**Figure.**
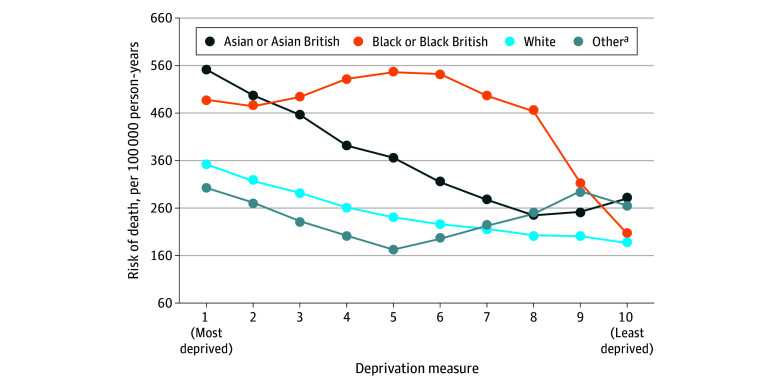
Risk of Deaths by Deprivation and by Race and Ethnicity (5-Point Smoothed) ^a^Defined as Arab or any other racial and ethnic groups not included.

In the unadjusted risk analysis, the RR of dying compared with White infants was higher for Black (RR, 1.93 [95% CI, 1.75-2.13]) and Asian (RR, 1.67 [95% CI, 1.55-1.80]) infants (Table 2; eFigure in Supplement 1). Multiracial infants (RR, 1.10 [95% CI, 0.98-1.23]) and infants of other races and ethnicities (RR, 0.98 [95% CI, 0.81-1.19]) had risks of death similar to that of White infants. The PAF for mortality of all infants with race and ethnicity other than White was 12.0% (95% CI, 10.3%-13.8%).

**Table 2.** Unadjusted Relative Risks of Infant Death by Race and Ethnicity, Stratified by Cause of Death (Imputed Dataset; N = 5621)

Measure	No.	Relative risk					PAF (95% CI), %
		White	Asian	Black	Multiracial	Other^a^	
Estimated No. of births	1 780 149	1 301 067	219 153	91 425	123 246	45 558	NA
Unadjusted relative risk							
All deaths	5621	1 [Reference]	1.67 (1.55 to 1.80)	1.93 (1.75 to 2.13)	1.10 (0.98 to 1.23)	0.98 (0.81 to 1.19)	12.0 (10.3 to 13.8)
Malignant neoplasm	NA	1 [Reference]	2.50 (1.23 to 5.07)	2.85 (1.11 to 7.32)	0.80 (0.19 to 3.40)	2.08 (0.49 to 8.79)	22.9 (3.1 to 42.6)
Preterm birth	NA	1 [Reference]	1.58 (1.40 to 1.78)	2.25 (1.94 to 2.61)	1.06 (0.88 to 1.27)	0.76 (0.54 to 1.07)	11.8 (8.9 to 14.6)
Intrapartum event	NA	1 [Reference]	1.04 (0.79 to 1.37)	1.29 (0.90 to 1.85)	0.93 (0.65 to 1.34)	0.93 (0.65 to 1.63)	1.3 (−4.0 to 6.6)
Infection	NA	1 [Reference]	1.57 (1.02 to 2.42)	1.75 (0.96 to 3.21)	1.16 (0.62 to 2.18)	0.87 (0.28 to 2.76)	11.2 (0.2 to 20.8)
Trauma	NA	1 [Reference]	0.64 (0.25 to 1.64)	1.21 (0.44 to 3.31)	0.84 (0.31 to 2.31)	0.63 (0.08 to 4.76)	−5.2 (−18.9 to 8.5)
SUDIC	NA	1 [Reference]	0.82 (0.64 to 1.05)	1.26 (0.94 to 1.68)	1.32 (1.03 to 1.68)	0.71 (0.41 to 1.22)	0.6 (−3.6 to 4.8)
Underlying disease	NA	1 [Reference]	2.63 (2.33 to 2.96)	2.21 (1.85 to 2.64)	1.09 (0.88 to 1.35)	1.47 (1.09 to 1.98)	21.9 (18.7 to 25.2)

Abbreviations: NA, not applicable; PAF, population attributable risk fraction; SUDIC, sudden unexpected death in infancy and childhood.

^a^ Defined as Arab or any other racial and ethnic groups.

Black infants had higher risks than White infants for dying from malignant neoplasms (RR, 2.85 [95% CI, 1.11-7.32]), preterm birth (RR, 2.25 [95% CI, 1.94-2.61]), and underlying disease (RR, 2.21 [95% CI, 1.85-2.64]) but similar risks for intrapartum events (RR, 1.29 [95% CI, 0.90-1.85]), infection (RR, 1.75 [95% CI, 0.96-3.21]), trauma (RR, 1.21 [95% CI, 0.44-3.31]), and sudden unexpected death in infancy and childhood (SUDIC) (RR, 1.26 [95% CI, 0.94-1.68]) (Table 2). Asian infants had higher risks than White infants for dying from malignant neoplasm (RR, 2.50 [95% CI, 1.23-5.07]), preterm birth (RR, 1.58 [95% CI, 1.40-1.78]), underlying disease (RR, 2.63 [95% CI, 2.33-2.96]), and infection (RR, 1.57 [95% CI, 1.02-2.42]). They had similar risks to White infants for death from intrapartum events (RR, 1.04 [95% CI, 0.79-1.37]), trauma (RR, 0.64 [95% CI, 0.25-1.64]), and SUDIC (RR, 0.82 [95% CI, 0.64-1.05]). Multiracial infants had higher risks than White infants for dying from SUDIC (RR, 1.32 [95% CI, 1.03-1.68]) but similar risks to White infants of death from malignant neoplasms, preterm birth, intrapartum events, infections, trauma, and underlying disease. Infants of other races and ethnicities had similar risks to White infants for all causes except underlying disease (RR, 1.47 [95% CI, 1.09-1.98]). Overall, if all infants had the same risks experienced by White infants, the PAFs of disease would be lower for malignant neoplasms (PAF, 22.9% [95% CI, 3.1%-42.6%]), preterm birth (PAF, 11.8% [95% CI, 8.9%-14.6%]), infections (PAF, 11.2% [95% CI, 0.2%-20.8%]), and underlying disease (PAF, 21.9% [95% CI, 18.7%-25.2%]) but similar for intrapartum events, trauma, and SUDIC.

There was some attenuation of the point estimates after adjusting for deprivation level (Table 3), although the RR of dying compared with White infants remained higher for Black (RR, 1.68 [95% CI, 1.52-1.87]) and Asian (RR, 1.52 [95% CI, 1.41-1.64]) infants. The PAF for all infant mortality also decreased slightly to 9.8% (95% CI, 8.0%-11.7%). After adjusting for gestational age at birth, the association of race and ethnicity with risk of death remained similar to the unadjusted analysis for all measures except preterm birth, where no clear association was seen for any racial and ethnic group (compared with White infants), with little evidence of a population-level association (PAF, −0.1% [95% CI, −3.3% to 3.2%]). As a consequence, the overall PAF decreased to 7.7% (95% CI, 5.9%-9.5%). After adjusting for region of England, the association of race and ethnicity with risk of death remained similar to the unadjusted analysis for all measures, with an overall PAF of 12.8% (95% CI, 11.0%-14.5%).

**Table 3.** Adjusted Relative Risks of Infant Death by Race and Ethnicity, Stratified by Cause of Death (Imputed Dataset; N = 5621)^a^

Measure	No.	Relative risk					PAF (95% CI), %
		White	Asian	Black	Multiracial	Other^b^	
**Adjusted for deprivation**							
All deaths	5621	1 [Reference]	1.52 (1.41 to 1.64)	1.68 (1.52 to 1.87)	1.07 (0.95 to 1.19)	0.90 (0.74 to 1.09)	9.8 (8.0 to 11.7)
Malignant neoplasm	NA	1 [Reference]	2.44 (1.19 to 4.99)	2.60 (0.97 to 6.98)	0.83 (0.20 to 3.52)	2.16 (0.51 to 9.15)	22.3 (2.4 to 42.3)
Preterm birth	NA	1 [Reference]	1.46 (1.29 to 1.65)	2.08 (1.79 to 2.43)	1.05 (0.87 to 1.26)	0.71 (0.50 to 1.00)	10.2 (7.3 to 13.1)
Intrapartum event	NA	1 [Reference]	1.04 (0.79 to 1.37)	1.28 (0.88 to 1.86)	0.95 (0.67 to 1.37)	0.95 (0.53 to 1.69)	1.4 (−3.9 to 6.8)
Infection	NA	1 [Reference]	1.52 (0.98 to 2.36)	1.58 (0.86 to 2.90)	1.22 (0.66 to 2.25)	0.82 (0.26 to 2.60)	9.9 (−0.7 to 20.5)
Trauma	NA	1 [Reference]	0.52 (0.18 to 1.50)	1.04 (0.38 to 2.85)	0.84 (0.30 to 2.32)	0.57 (0.08 to 4.10)	−9.0 (−23.1 to 5.2)
SUDIC	NA	1 [Reference]	0.69 (0.54 to 0.88)	0.99 (0.72 to 1.31)	1.29 (1.01 to 1.64)	0.61 (0.36 to 1.03)	−3.6 (−8.0 to 0.8)
Underlying disease	NA	1 [Reference]	2.35 (2.08 to 2.65)	1.83 (1.52 to 2.20)	1.04 (0.84 to 1.29)	1.34 (1.00 to 1.81)	19.3 (15.9 to 22.7)
**Adjusted for gestational age at birth**							
All deaths	5621	1 [Reference]	1.45 (1.35 to 1.56)	1.27 (1.15 to 1.41)	1.07 (0.96 to 1.20)	0.90 (0.73 to 1.10)	7.7 (5.9 to 9.5)
Malignant neoplasm	NA	1 [Reference]	2.35 (1.16 to 4.76)	2.17 (0.82 to 5.73)	0.86 (0.20 to 3.61)	2.24 (0.53 to 9.48)	21.6 (1.4 to 41.7)
Preterm birth^c^	NA	1 [Reference]	1.13 (1.00 to 1.28)	0.99 (0.85 to 1.15)	0.95 (0.79 to 1.14)	0.54 (0.36 to 0.79)	0.3 (−3.0 to 3.5)
Intrapartum event	NA	1 [Reference]	1.05 (0.80 to 1.38)	1.15 (0.79 to 1.66)	1.00 (0.70 to 1.44)	1.01 (0.57 to 1.79)	1.5 (−3.7 to 6.8)
Infection	NA	1 [Reference]	1.66 (1.07 to 2.55)	1.73 (0.95 to 3.14)	1.30 (0.70 to 2.39)	0.90 (0.28 to 2.93)	12.0 (1.7 to 22.2)
Trauma	NA	1 [Reference]	0.60 (0.21 to 1.72)	1.23 (0.45 to 3.32)	0.89 (0.32 to 2.46)	0.68 (0.09 to 4.90)	−5.2 (−18.6 to 8.2)
SUDIC	NA	1 [Reference]	0.79 (0.62 to 1.01)	1.22 (0.92 to 1.62)	1.40 (1.10 to 1.78)	0.75 (0.45 to 1.27)	0.06 (−3.6 to 4.8)
Underlying disease	NA	1 [Reference]	2.53 (2.24 to 2.85)	1.99 (1.66 to 2.38)	1.12 (0.90 to 1.39)	1.56 (1.16 to 2.11)	21.3 (18.0 to 24.5)
**Adjusted for region of England**							
All deaths	5621	1 [Reference]	1.69 (1.57 to 1.83)	2.09 (1.88 to 2.32)	1.15 (1.03 to 1.29)	1.04 (0.86 to 1.26)	12.8 (11.0 to 14.5)
Malignant neoplasm	NA	1 [Reference]	2.55 (1.22 to 5.31)	2.75 (1.00 to 7.55)	0.88 (0.21 to 3.73)	2.25 (0.52 to 9.63)	23.2 (3.2 to 43.2)
Preterm birth	NA	1 [Reference]	1.58 (1.39 to 1.79)	2.51 (2.15 to 2.94)	1.11 (0.92 to 1.34)	0.81 (0.57 to 1.15)	12.6 (9.7 to 15.4)
Intrapartum event	NA	1 [Reference]	1.03 (0.78 to 1.36)	1.24 (0.85 to 1.81)	0.94 (0.66 to 1.36)	0.93 (0.52 to 1.66)	1.0 (−4.5 to 6.5)
Infection	NA	1 [Reference]	1.79 (1.14 to 2.80)	1.96 (1.06 to 3.64)	1.33 (0.72 to 2.47)	0.96 (0.30 to 3.07)	13.5 (3.1 to 23.8)
Trauma	NA	1 [Reference]	0.63 (0.22 to 1.84)	1.53 (0.55 to 4.25)	0.96 (0.34 to 2.69)	0.75 (0.10 to 5.46)	−2.7 (−16.2 to 10.9)
SUDIC	NA	1 [Reference]	0.85 (0.66 to 1.09)	1.42 (1.06 to 1.90)	1.46 (1.14 to 1.86)	0.79 (0.47 to 1.33)	2.6 (−1.6 to 6.8)
Underlying disease	NA	1 [Reference]	2.62 (2.31 to 2.96)	2.27 (1.88 to 2.74)	1.12 (0.90 to 1.39)	1.55 (1.15 to 2.09)	22.0 (18.7 to 25.3)

Abbreviations: NA, not applicable; PAF, population attributable risk fraction; SUDIC, sudden unexpected death in infancy and childhood.

^a^ Adjusted for deprivation, gestational age at birth, or region of England.

^b^ Defined as Arab or any other racial and ethnic groups.

^c^ Restricted to birth less than 37 weeks’ gestational age.

A total of 515 deaths occurred from underlying conditions after May 1, 2021. Most were due to either congenital heart disease or other chromosomal or congenital anomalies (99.0% [487 of 492]) (eTable 3 in Supplement 1), and for both and in all models, there was evidence of an increased PAF, using White infants as the baseline reference group. The number of deaths among infants of other races and ethnicities (n = 5) was too small to adequately quantify. Results from the complete case analysis were compatible with the main analysis (eTable 4 in Supplement 1).

## Discussion

The race and ethnicity of infants who died before the age of 1 year is patterned across the regions of England, level of deprivation, and gestational age at birth. Overall, if all infants in England had the same risk of death as White infants, 12.0% of deaths would be avoided (>200 infants per year), with deaths from malignant neoplasms, preterm birth, infection, and underlying disease having the highest associations with this outcome. These estimates were attenuated slightly after adjustment for the local level of deprivation, although the overall PAF remained high at 9.8%. However, much of the PAF may be explained by the gestational age at which the infants were born because after adjustment for gestational age, the PAF decreased to 7.7%, with the data suggesting that survival after preterm birth is similar across racial and ethnic groups. However, the association of race and ethnicity with deprivation (in particular) is complex, with the risk profile appearing linear for some groups (ie, White and Asian infants) but not so clear for Black infants, where the risk remained substantially higher than for other groups except for children living in the most affluent areas.

Infant mortality, a key measure of a society’s overall health and well-being, remains a difficult global challenge marked by significant inequalities. Disparities in infant mortality rates persist across countries, regions, and socioeconomic strata, reflecting a complex interplay of several factors.^20,21,22^ Inequalities in infant mortality have a profound impact, albeit in different ways, in low-income and high-income countries. In low-income countries, where resources are limited and the health care infrastructure often inadequate, the association of inequalities with infant mortality is particularly harmful, with much bigger impacts than seen in this work.^23^ Disparities in access to quality health care services, including prenatal care, skilled birth attendants, and essential medical interventions, are associated with higher infant mortality rates among disadvantaged populations. These inequalities perpetuate a vicious cycle of poverty and poor health outcomes, hindering overall socioeconomic development.

In high-income countries, although overall infant mortality rates tend to be lower, inequalities still persist. Although most work is unable to integrate the patterning of race and ethnicity and deprivation, this work is consistent with older work based on 2006-2012 births in the UK and Wales, which reported higher overall infant mortality for a number of races and ethnicities other than White race, and it concluded that higher infant mortality in South Asian and Black infants, in particular, did not appear to be explained by sociodemographic characteristics.^24^ However, whether this is due to health behaviors around access and attendance to antenatal screening is unclear at present.^25^ Deaths by SUDIC are particularly interesting, with the lowest risks among Asian infants (and perhaps infants of other ethnicities). Differences in modifiable factors, such as smoking or other health behaviors (such as unsafe sleeping practices), may underpin these differences.^13^ Risk of death from infection also appeared to be patterned by race and ethnicity, which was seen across all analyses. Although some of these deaths may be confounded by underlying (but not fatal) congenital anomalies or genetic disease, the potential to avoid some of these deaths raises concerns over early recognition of sepsis and access to health care for some racial and ethnic minority groups.^26^

Although the broad association of deprivation with child mortality remains important, this work suggests it does not explain the excess child mortality seen in some racial and ethnic groups, which is concerning considering our context of universally funded health and social care in England. Although some residual confounding undoubtedly exists, the lack of a measurable shift in the point estimates is striking. Perinatal events also represent the biggest number of childhood deaths in England,^27,28^ and efforts to reduce the number of deaths could have profound effects. This analysis does, however, suggest that in England, the mortality from prematurity may be associated with the risk of preterm birth, suggesting that preterm birth may, in part, mediate the association of race and ethnicity with infant mortality.

Efforts to engage and support women at risk with enhanced antenatal care and to address identified barriers should be seen as a priority. Delivery of health care around birth may be affected by language and engagement^28,29^ in acute decision-making; a new task force in the UK to explore inequalities in maternity care may address some of these risks.^30^ However, race and ethnicity are also closely associated with migration, refugee status, and asylum seeking; some of the association here may reflect the vulnerabilities and complex needs of this group.^31^ Although perinatal death and preterm death were associated with overall childhood mortality, preterm births did not explain the overall increased mortality in any single group.^24^

Recent independent national reviews have reported inequity of access, experience, and health outcomes in the NHS for people from minority racial and ethnic groups associated with systemic racism and compromised communication as a consequence of language barriers.^32,33^ Overall, the review of maternal health care found evidence of negative interactions, stereotyping, disrespect, discrimination, and cultural insensitivity, resulting in suboptimal engagement and pregnancy care. The recommendations were to ensure ready access to high-quality interpreting and translation services, a commitment to address racist attitudes and behaviors among staff, and to address structural dimensions of NHS systems that discriminate against women from racial and ethnic minority groups and their infants. A further systematic scoping review of maternal and newborn health in England by the NHS Race and Health Observatory found a lack of detailed policy to address racial and ethnic health inequalities, and it concluded that interventions targeting institutional and interpersonal racism at the organizational level are urgently needed,^34^ in contrast to the findings of a government-commissioned report dismissing structural racism as a cause of poor health outcomes during the COVID-19 pandemic.^12,35^ However, in other work, health inequalities may not be strongly associated with overall country-level gross domestic product or even health expenditure but may be associated with broader government spending,^23^ suggesting that investment into wider areas of social support, employment, and infrastructure may be vital in reducing the health outcome inequities seen here, and elsewhere, with racial discrimination being a global risk factor for disease.^36^

### Limitations

This study has some limitations. As with any routine data linkage, some data on the exposure (eg, race and ethnicity) and covariates (eg, gestational age) are missing, which should be considered when interpreting this work alongside assumptions about the models used. However, the work is based on statutory data reported to the NCMD, and previous work has shown good validation and coverage.^37,38^ Race and ethnicity data were available for 92% of deaths reported to the NCMD, and the main analysis used a multiple imputation technique to minimize bias (and provided compatible results with a complete-case analysis). A further limitation is that the population at risk was derived from ONS birth registrations in 2019 and applied across the 3 years of the data. Risk was therefore derived assuming a similar number and profile of births across the period. Although it seems unlikely that substantial changes occurred during this time, any interpretation should bear this in mind. The provisional category of death used in the analysis is based on information at the 48-hour notification and may be different from the final cause after all investigations (particularly for deaths defined as SUDIC),^39^ while a higher number of deaths in one group (ie, preterm birth) may mean consequential lower risks in other groups (eg, SUDIC); however, the main interpretation of this work should be about overall infant mortality, and given the very low individual risk of death, we feel a wider, cautious interpretation is acceptable.

In addition, we were only able to adjust for covariates separately to assess possible causal pathways, and for our measures of deprivation, this was done on a local area measure rather than a family-level measure of income and education, so some residual confounding is likely. However, while the causal pathways are likely complex, the real risk faced by infants in England should be taken from the unadjusted estimates. The main limitation of this work is likely to be the precision of the estimates, with childhood death a rare event, and 95% CIs were wide for some of the stratified results and may include important differences for rarer events (eg, intrapartum events).

## Conclusions

This cohort study found that the proportion of infants who die at younger than 1 year of age is patterned by their race and ethnicity, with a PAF of 12.0% among all infants who are not White, compared with White infants. Avoidance of 12% of infant deaths in England would attain an infant mortality rate of 3.3 per 1000 live births, matching the latest European Union mean rate.^40^ An overconservative adjustment for deprivation did not explain the patterns seen, with higher risks of death after preterm birth, malignant neoplasms, infection, and underlying disease. Deaths after intrapartum events, trauma, and SUDIC appeared less patterned by the infants’ race and ethnicity. Although the causal processes need to be clarified, racial and ethnic disparities in mortality are not due to biology but rather to a complex interaction of factors in the sociocultural environment. Approximately half of the PAF may be due to increased risk of preterm birth in Asian and Black communities, and work is needed to identify what can be done to reduce this. Once preterm birth has occurred, infant deaths appear to not be associated with race and ethnicity.

## Notes

Supplement 1.
eMethods.
**eTable 1.** Multiple Imputation Model **eTable 2.** Characteristics of Infant Deaths Reported to NCMD in England Between April 2019 and March 2022; Split by Ethnicity **eTable 3.** Number and RR of Infant Death by Ethnicity, Stratified by Subcategory of Underlying Disease (Deaths Between May 1, 2021, and March 2022) (n=515 Deaths) **eTable 4.** Unadjusted, and Adjusted RR of Infant Death by Ethnicity, Stratified by Cause of Death: Complete Case Analysis **eFigure.** Population Attributable Risk Fractions for All-Cause Infant Deaths, and Cause-Specific Deaths; Split by Analysis Model

Supplement 2.
Data Sharing Statement
