# Supplementary material for: Race and Ethnicity, Deprivation, and Infant Mortality in England, 2019-2022
Source: JAMA Netw Open. 2024 Feb 12;7(2):e2355403. doi: 10.1001/jamanetworkopen.2023.55403 (PMC10862146; doi:10.1001/jamanetworkopen.2023.55403)
Supplement: Supplement 1. — eMethods. eTable 1. Multiple Imputation Model eTable 2. Characteristics of Infant Deaths Reported to NCMD in England Between April 2019 and March 2022; Split by Ethnicity eTable 3. Number and RR of Infant Death by Ethnicity, Stratified by Subcategory of Underlying Disease (Deaths Between May 1, 2021, and March 2022) (n=515 Deaths) eTable 4. Unadjusted, and Adjusted RR of Infant Death by Ethnicity, Stratified by Cause of Death: Complete Case Analysis eFigure. Population Attributable Risk Fractions for All-Cause Infant Deaths, and Cause-Specific Deaths; Split by Analysis Model [file jamanetwopen-e2355403-s001.pdf]

Supplementary Online Content

Odd DE, Stoianova S, Williams T, et al. Race and ethnicity, deprivation, and infant mortality in England, 2019-2022. *JAMA Netw Open.* 2024;7(2):e2355403. doi:10.1001/jamanetworkopen.2023.55403

eMethods.

eTable 1. Multiple Imputation Model

eTable 2. Characteristics of Infant Deaths Reported to NCMD in England Between April 2019 and March 2022; Split by Ethnicity

eTable 3. Number and RR of Infant Death by Ethnicity, Stratified by Subcategory of Underlying Disease (Deaths Between May 1, 2021, and March 2022) (n=515 Deaths)

eTable 4. Unadjusted, and Adjusted RR of Infant Death by Ethnicity, Stratified by Cause of Death: Complete Case Analysis

eFigure. Population Attributable Risk Fractions for All-Cause Infant Deaths, and Cause-Specific Deaths; Split by Analysis Model

This supplementary material has been provided by the authors to give readers additional information about their work.

© 2024 Odd DE et al. *JAMA Network Open.*

eMethods.

Data, Study Design and Population

The National Child Mortality Database (NCMD) Programme collates and analyses data on all children in England who die before their 18th birthday; with statutory death notification within 48 hours of death from the 58 Child Death Overview Panels (CDOPs) in England.

Outcome

All coders recorded a provisional category of death (see below) or that there was insufficient information provided. For each death, if two or more coders agreed on a category this was taken as the most likely category and where no two coders agreed, the category highest in the following hierarchy was used (based on categorisation used by CDOPs, in order of priority: Trauma, Malignancy, Underlying Medical Condition, Intrapartum event, Preterm Birth, Infection, SUDIC (Sudden Unexpected Death in Infant and Childhood). In addition, for deaths after 1st May 2022, further details were derived for those deaths from Underlying Medical Conditions, to further categorise them as Cardiac (congenital), Cardiac (acquired), Other chromosomal or congenital abnormality, Acute medical condition Chronic medical condition, or Other condition.

Confounders

The Index of Multiple Deprivation is derived at the resolution of the Lower Super Output Area (LSOA) and is a measure derived from 10 sub-domains (Income, Employment, Child education, Adult education, Health, Crime, Geographic Barriers, Wider barriers, Outdoor living environment, and Indoor living environment) derived for each area containing between 1000 to 3000 people. Data on the sex of individual (female, male, other (including not known)) was obtained from the CDOP notification.

Statistical Analysis

Multiple imputation was performed using the Stata ICE program, to impute missing values of ethnicity, gestation, provisional category of death and deprivation. Twenty datasets were imputed and regression analysis repeated, see eTable 1 for further details. Point estimates and measures of uncertainty were combined using Rubins rules to derive final measures.

Population attributable risk fraction (PAF) was derived using the Stata command ‘punaf’.

Where frequency counts were below 5, or could be derived, absolute numbers are not presented. Analysis was performed using Stata version 17. All measures had 95% confidence intervals derived.

© 2024 Odd DE et al. *JAMA Network Open.*

eTable 1. Multiple Imputation Model

Variable	Imputation command	% missing
Ethnicity	mlogit	472 (8·4%)
Year of Birth	-	0 (0·0%)
Index of Multiple Deprivation	-	0 (0·0%)
Rural or Urban area	-	0 (0·0%)
Cause of death	mlogit	147 (2·6%)
Sex	logit	43 (0·8%)
Region of England	-	0 (0·0%)
Gestational age at birth	ologit	204 (3·6%)

© 2024 Odd DE et al. *JAMA Network Open.*

eTable 2. Characteristics of Infant Deaths Reported to NCMD in England Between April 2019 and March 2022; Split by Ethnicity

Measure	N	White	Asian	Black	Multi-racial	Other*	p-value
Estimate Number of Births	1,780,149	1,301,067	219,153	91,425	123,246	45,558	
All Deaths	5149	3318 (64.4%)	927 (18.0%)	448 (8.7%)	343 (6.6%)	113 (2.2%)	
Sex	5130						0.534
Female		1463 (44.2%)	415 (44.9%)	214 (48.1%)	150 (44.1%)	46 (40.7%)	
Male		1845 (55.8%)	509 (55.1%)	231 (51.9%)	190 (55.9%)	67 (59.3%)	
Gestational Age	4989	33 (25-38)	34 (25-38)	30 (24-37)	34 (25-38)	35 (26-38)	0.009
Region	5149						<0.001
East Midlands		310 (9.3%)	64 (6.9%)	23 (5.1%)	33 (9.6%)	-	
East of England		348 (10.5%)	54 (5.8%)	31 (6.9%)	39 (11.4%)	10 (8.9%)	
London		347 (10.5%)	249 (26.9%)	203 (45.3%)	66 (19.2%)	30 (26.6%)	
North East		196 (5.9%)	23 (2.5%)	-	-	-	
North West		505 (15.2%)	136 (14.7%)	37 (8.3%)	28 (8.2%)	14 (12.4%)	
South East		494 (14.9%)	77 (8.3%)	46 (10.3%)	56 (16.3%)	9 (8.0%)	
South West		325 (9.8%)	15 (1.6%)	11 (2.5%)	24 (7.0%)	8 (7.1%)	
West Midlands		423 (12.8%)	184 (19.9%)	66 (14.7%)	63 (18.4%)	24 (21.2%)	
Yorkshire and the Humber		370 (11.2%)	125 (13.5%)	27 (6.0%)	32 (9.3%)	10 (8.9%)	
Deprivation Decile	5149						<0.001
1/2 (Most Deprived)		1024 (30.9%)	431 (46.5%)	192 (42.9%)	122 (35.6%)	50 (44.3%)	
3/4		734 (22.1%)	250 (27.0%)	139 (31.0%)	88 (25.7%)	29 (25.7%)	
5/6		605 (18.2%)	138 (14.9%)	75 (16.7%)	54 (15.7%)	12 (10.6%)	
7/8		512 (15.4%)	58 (6.3%)	36 (8.0%)	46 (13.4%)	11 (9.7%)	
9/10 (Least Deprived)		443 (13.4%)	50 (5.4%)	6 (1.3%)	33 (9.6%)	11 (9.7%)	

Values are number (%)

* Other is Arab or any other ethnic group.

© 2024 Odd DE et al. *JAMA Network Open.*

eTable 3. Number and RR of Infant Death by Ethnicity, Stratified by Subcategory of Underlying Disease (Deaths Between May 1, 2021, and March 2022) (n=515 Deaths)

**Measure**				**Relative Risk (vs White ethnicity)**			
	**n**	**White**	**Asian**	**Black**	**Multi-racial**	**Other***	**Population Attributable Risk Fraction (PAF (95% CI))**
Estimate Number of Births	542,779	397,548	66,963	27,935	37,659	12,674	
**Number of Deaths**	492	n (%)	n (%)	n (%)	n (%)	n (%)	
Congenital Heart Disease	149	96 (34.0%)	28 (22.6%)	12 (30.0%)	7 (21.9%)	6 (42.9%)	
Other Chromosomal or Congenital	338	183 (64.9%)	95 (76.6%)	27 (67.5%)	25 (78.1%)	8 (57.1%)	
Other	5	-	-	-	-	-	
**Relative Risks**							
**Unadjusted Relative Risk**	515	**RR (95% CI)**	**RR (95% CI)**	**RR (95% CI)**	**RR (95% CI)**	**RR (95% CI)**	
Congenital Heart Disease		1 (Ref)	1.76 (1.16-2.66)	1.75 (0.96-3.19)	0.83 (0.38-1.80)	3.80 (1.60-8.99)	13.4% (3.3% to 23.5%)
Other Chromosomal or Congenital		1 (Ref)	3.11 (2.43-3.98)	2.14 (1.43-3.98)	1.43 (0.94-2.18)	2.72 (1.26-5.86)	27.2% (20.1% to 34.3%)
Other		1 (Ref)	1.85 (0.20-16.78)	3.73 (0.40-34.40)	-	-	20.5% (-35.2% to 75.2%)
**Adjusted for Deprivation**							
Congenital Heart Disease		1 (Ref)	2.37 (1.55-3.62)	2.88 (1.55-5.37)	0.91 (0.42-1.98)	3.56 (1.54-8.21)	18.3% (8.8% to 27.9%)
Other Chromosomal or Congenital		1 (Ref)	4.70 (3.66-6.05)	4.36 (2.87-6.61)	1.63 (1.07-2.48)	2.61 (1.27-5.26)	32.6% (26.0% to 39.2%)
Other		1 (Ref)	2.39 (0.25-22.87)	5.95 (0.58-60.68)	-	-	24.3% (-28.2% to 76.8%)
**Adjusted for gestation at birth**							
Congenital Heart Disease		1 (Ref)	1.84 (1.22-2.79)	2.02 (1.10-3.68)	0.90 (0.41-1.95)	2.25 (0.99-5.13)	14.2% (4.3% to 24.2%)
Other Chromosomal or Congenital		1 (Ref)	3.37 (2.56-4.18)	2.41 (1.61-3.60)	1.59 (1.05-2.42)	1.73 (0.84-3.54)	28.1% (21.1% to 35.0%)
Other		1 (Ref)	1.93 (0.21-17.56)	4.41 (0.48-40.71)	-	-	20.6% (-34.1% to 75.3%)
**Adjusted for region of England**							
Congenital Heart Disease		1 (Ref)	1.76 (1.15-2.69)	1.89 (1.01-3.52)	0.86 (0.39-1.89)	1.96 (0.84-4.57)	12.9% (2.6% to 23.3%)
Other Chromosomal or Congenital		1 (Ref)	3.25 (2.51-4.20)	2.45 (1.61-3.71)	1.61 (1.05-2.45)	1.48 (0.72-3.06)	27.8% (20.7% to 35.0%)
Other		1 (Ref)	2.66 (0.27-25.70)	5.75 (0.52-63.81)	-	-	25.6% (-26.8% to 77.9%)

Values are relative risk (95% CI) or population attributable risk fraction (95% CI)

* Other is Arab or any other ethnic group.

© 2024 Odd DE et al. *JAMA Network Open.*

eTable 4. Unadjusted, and Adjusted RR of Infant Death by Ethnicity, Stratified by Cause of Death: Complete Case Analysis

Measure	n	Relative Risk (vs White ethnicity)					
		White	Asian	Black	Multi-racial	Other*	PAF (95% CI)
Estimate Number of Births	1,780,149	1,301,067	219,153	91,425	123,246	45,558	-
**Unadjusted Relative Risk**							
All Deaths	5149	1 (Ref)	1.66 (1.54-1.78)	1.92 (1.74-2.12)	1.09 (0.98-1.22)	0.97 (0.81-1.17)	11.8% (10.0% to 13.6%)
Malignancy		1 (Ref)	2.72 (1.33-5.55)	2.96 (1.13-7.77)	0.88 (0.21-3.72)	2.38 (0.56-10.07)	25.4% (2.2% to 43.0%)
Preterm Birth		1 (Ref)	1.56 (1.38-1.76)	2.29 (1.96-2.66)	1.05 (0.88-1.26)	0.75 (0.53-1.06)	11.7% (8.8% to 14.6%)
Intrapartum Event		1 (Ref)	1.02 (0.78-1.34)	1.26 (0.88-1.82)	0.94 (0.65-1.35)	0.95 (0.54-1.69)	1.1% (-4.5% to 6.3%)
Infection		1 (Ref)	1.61 (1.04-2.48)	1.78 (0.98-3.24)	1.21 (0.65-2.26)	0.89 (0.28-2.82)	11.2% (0.0% to 21.1%)
Trauma		1 (Ref)	0.47 (0.17-1.32)	1.14 (0.41-3.15)	0.84 (0.31-2.34)	0.57 (0.08-4.13)	-8.6% (-23.2% to 4.2%)
SUDIC		1 (Ref)	0.74 (0.58-0.94)	1.23 (0.92-1.64)	1.37 (1.08-1.74)	0.69 (0.41-1.17)	-0.3% (-4.6% to 3.8%)
Underlying Disease		1 (Ref)	2.68 (2.38-3.02)	2.18 (1.82-2.61)	1.07 (0.86-1.33)	1.50 (1.11-2.02)	22.2% (18.7% to 25.5%)
**Adjusted for Deprivation**							
All Deaths	5149	1 (Ref)	1.51 (1.40-1.63)	1.67 (1.51-1.85)	1.06 (0.95-1.18)	0.88 (0.73-1.06)	9.6% (7.7% to 11.4%)
Malignancy		1 (Ref)	2.67 (1.30-5.51)	2.88 (1.08-7.68)	0.87 (0.20-3.68)	2.32 (0.54-9.86)	24.8% (1.3% to 42.7%)
Preterm Birth		1 (Ref)	1.46 (1.29-1.65)	2.07 (1.78-2.41)	1.03 (0.86-1.24)	0.70 (0.49-0.99)	10.0% (7.0% to 13.0%)
Intrapartum Event		1 (Ref)	1.03 (0.78-1.35)	1.28 (0.89-1.85)	0.94 (0.66-1.35)	0.96 (0.54-1.70)	1.2% (-4.4% to 6.5%)
Infection		1 (Ref)	1.51 (0.98-2.35)	1.63 (0.88-2.99)	1.18 (0.63-2.21)	0.84 (0.27-2.66)	9.8% (1.7% to 20.0%)
Trauma		1 (Ref)	0.43 (0.15-1.20)	0.99 (0.35-2.78)	0.83 (0.30-2.31)	0.51 (0.07-3.69)	-11.3% (-26.6% to 2.2%)
SUDIC		1 (Ref)	0.62 (0.49-0.80)	0.97 (0.72-1.29)	1.29 (1.01-1.64)	0.58 (0.34-0.99)	-4.8% (-9.3% to -0.4%)
Underlying Disease		1 (Ref)	2.41 (2.13-2.71)	1.84 (1.53-2.22)	1.03 (0.83-1.28)	1.34 (0.99-1.80)	19.8% (16.2% to 23.3%)
**Adjusted for gestation at birth**							
All Deaths	4989	1 (Ref)	1.46 (1.36-1.57)	1.28 (1.16-1.42)	1.07 (0.95-1.19)	0.88 (0.72-1.07)	7.8% (5.9% to 9.7%)
Malignancy		1 (Ref)	2.77 (1.25-6.15)	3.48 (1.30-9.35)	0.57 (0.08-4.28)	3.06 (0.71-13.14)	28.2% (2.3% to 47.3%)*
Preterm Birth		1 (Ref)	1.12 (0.99-1.27)	0.97 (0.83-1.12)	0.92 (0.77-1.11)	0.51 (0.35-0.75)	-0.3% (-3.7% to 2.9%)
Intrapartum Event		1 (Ref)	1.03 (0.78-1.36)	1.18 (0.82-1.69)	1.01 (0.70-1.45)	1.05 (0.59-1.86)	1.5% (-4.1% to 6.8%)
Infection*		1 (Ref)	1.57 (1.00-2.47)	1.71 (0.93-3.14)	1.25 (0.65-2.40)	0.68 (0.17-2.76)	12.4% (0.7% to 22.7%)*
Trauma		1 (Ref)	0.39 (0.12-1.26)	0.89 (0.28-2.87)	0.97 (0.35-2.68)	0.66 (0.09-4.78)	-9.3% (-24.4% to 4.0%)*
SUDIC		1 (Ref)	0.73 (0.57-0.94)	1.26 (0.93-1.69)	1.42 (1.10-1.82)	0.78 (0.46-1.32)	0.2% (-4.3% to 4.6%)
Underlying Disease		1 (Ref)	2.66 (2.36-3.00)	2.06 (1.72-2.48)	1.12 (0.90-1.40)	1.52 (1.12-2.06)	22.4% (18.9% to 25.7%)
**Adjusted for region of England**	5149						
All Deaths		1 (Ref)	1.69 (1.57-1.82)	2.08 (1.88-2.31)	1.14 (1.02-2.31)	1.03 (1.02-1.28)	12.6% (10.8% to 14.4%)
Malignancy		1 (Ref)	2.88 (1.37-6.06)	3.06 (1.12-8.39)	0.91 (0.21-3.89)	2.46 (0.57-10.62)	25.9% (2.4% to 43.7%)
Preterm Birth		1 (Ref)	1.57 (1.39-1.78)	2.48 (2.12-2.89)	1.09 (0.91-1.31)	0.80 (0.56-1.13)	12.3% (9.3% to 15.2%)
Intrapartum Event		1 (Ref)	1.01 (0.77-1.34)	1.23 (0.85-1.78)	0.93 (0.64-1.34)	0.94 (0.52-1.67)	0.6% (-5.2% to 6.1%)
Infection		1 (Ref)	1.77 (1.13-2.77)	1.99 (1.07-3.71)	1.29 (0.69-2.42)	0.98 (0.31-3.12)	13.2% (1.9% to 23.2%)
Trauma		1 (Ref)	0.53 (0.19-1.47)	1.50 (0.53-4.24)	0.97 (0.35-2.71)	0.69 (0.09-5.02)	-4.6% (-19.1% to 8.2%)
SUDIC		1 (Ref)	0.78 (0.61-1.00)	1.39 (1.03-1.87)	1.46 (1.14-1.86)	0.75 (0.44-1.28)	1.6% (-2.8% to 5.8%)
Underlying Disease		1 (Ref)	2.72 (2.41-3.08)	2.35 (1.95-2.84)	1.12 (0.90-1.40)	1.57 (1.17-2.12)	22.8% (19.3% to 26.2%)

Values are relative risk (95% CI) or population attributable risk fraction (95% CI)
* Other is Arab or any other ethnic group.

© 2024 Odd DE et al. *JAMA Network Open.*

eFigure. Population Attributable Risk Fractions for All-Cause Infant Deaths, and Cause-Specific Deaths; Split by Analysis Model

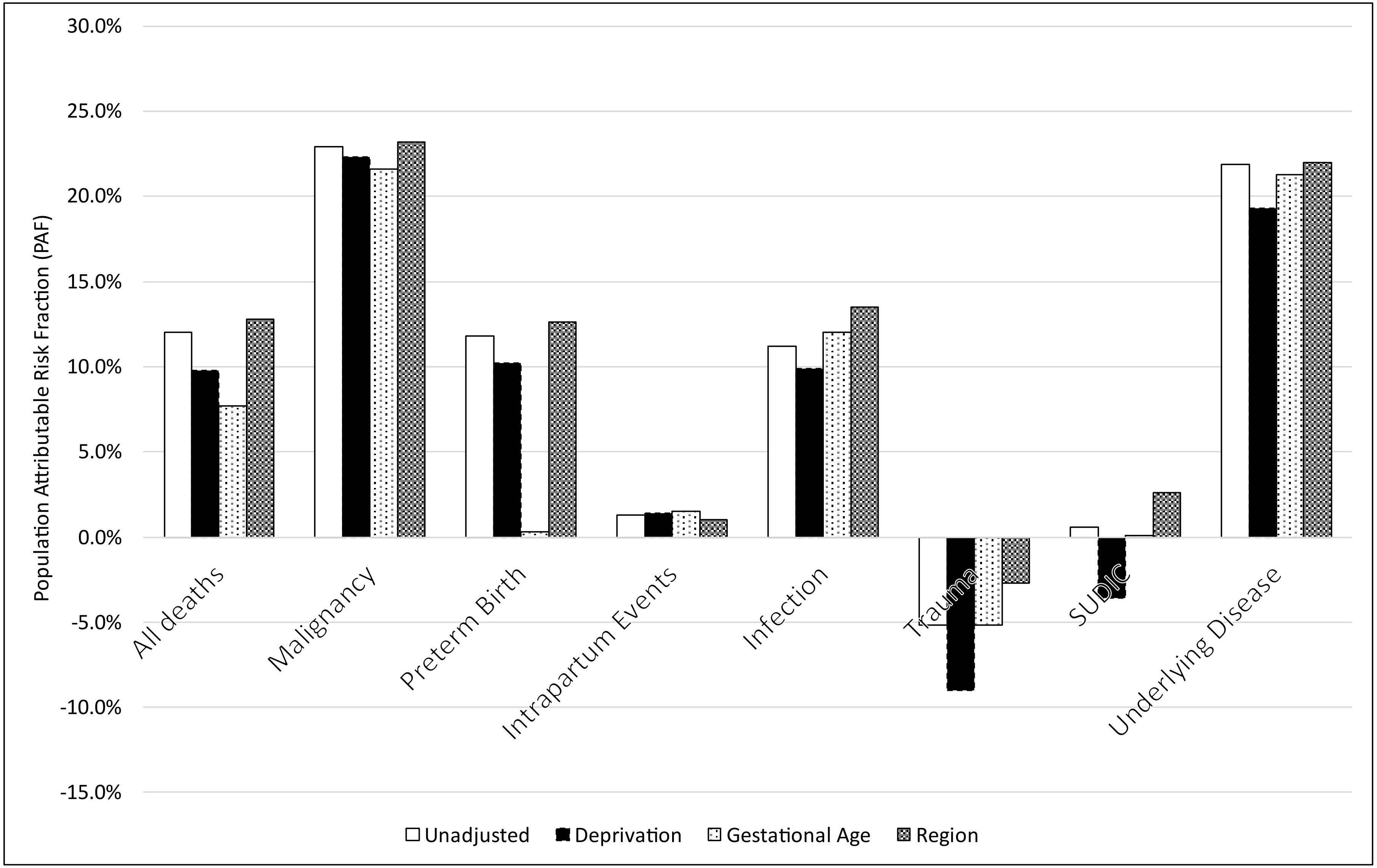

© 2024 Odd DE et al. *JAMA Network Open.*
