# Supplementary material for: Race and Ethnicity, Deprivation, and Infant Mortality in England, 2019-2022
Source: JAMA Netw Open. 2024 Feb 12;7(2):e2355403. doi: 10.1001/jamanetworkopen.2023.55403 (PMC10862146; doi:10.1001/jamanetworkopen.2023.55403)
Supplement: Supplement 2. — Data Sharing Statement [file jamanetwopen-e2355403-s002.pdf]

Data Sharing Statement

Odd. Race and Ethnicity, Deprivation, and Infant Mortality in England, 2019-2022. *JAMA Netw Open*. Published February 12, 2024. doi:10.1001/jamanetworkopen.2023.55403

Data
Data available: No

Additional Information
Explanation for why data not available: The legal basis for data collection does not permit extended use of the data
